# The association between benzodiazepine prescriptions and the risk of laxative use in schizophrenia treatment

**DOI:** 10.1002/npr2.12499

**Published:** 2024-11-07

**Authors:** Shinichiro Ochi, Takashi Tsuboi, Naomi Hasegawa, Hikaru Hori, Kayo Ichihashi, Yayoi Imamura, Tsuyoshi Okada, Fumitoshi Kodaka, Yoshitaka Saito, Jun‐ichi Iga, Toshiaki Onitsuka, Kiyokazu Atake, Shu‐ichi Ueno, Ryota Hashimoto, Norio Yasui‐Furukori

**Affiliations:** ^1^ Department of Neuropsychiatry, Molecules and Function Ehime University Graduate School of Medicine Ehime Japan; ^2^ Department of Neuropsychiatry Kyorin University School of Medicine Tokyo Japan; ^3^ Department of Pathology of Mental Diseases National Institute of Mental Health, National Center of Neurology and Psychiatry Tokyo Japan; ^4^ Department of Psychiatry, Faculty of Medicine Fukuoka University Fukuoka Japan; ^5^ Department of Neuropsychiatry University of Tokyo Hospital Tokyo Japan; ^6^ Department of Psychiatry Jichi Medical University Shimotsuke Japan; ^7^ Department of Psychiatry The Jikei University School of Medicine Tokyo Japan; ^8^ Department of Psychiatry Kitasato University School of Medicine Sagamihara Japan; ^9^ National Hospital Organization Sakakibara National Hospital Tsu Japan; ^10^ Nippon Telegraph and Telephone West Corporation Kyushu Health Administration Center Fukuoka Japan; ^11^ Department of Psychiatry Dokkyo Medical University School of Medicine Tochigi Japan

**Keywords:** anti‐cholinergic, benzodiazepine, constipation, EGUIDE, laxative

## Abstract

**Aim:**

Constipation is one of the most common adverse effects in schizophrenia treatment, and it can sometimes cause severe gastrointestinal disease. However, the results of association studies between constipation and psychotropic medications in patients with schizophrenia are inconsistent. Therefore, we investigated the characteristics of psychotropic and laxative prescriptions at discharge in patients with schizophrenia to clarify the association between psychotropics and constipation.

**Methods:**

We analyzed the data of 139 patients with schizophrenia with or without laxative prescriptions at discharge from eight institutions in 2020.

**Results:**

Sixty‐two patients were prescribed laxatives at discharge. The prescription of benzodiazepines in the laxative use group (66.1%) was significantly higher than that in the non‐laxative use group (39.0%) (*p* = 1.4 × 10^−3^), and the mean number of benzodiazepines in the laxative use group (1.2 ± 1.1/day) was significantly higher than that in the non‐laxative use group (0.7 ± 0.9/day) (*p* = 2.6 × 10^−3^). Multivariate logistic regression analyses revealed that benzodiazepine prescriptions were significantly associated with laxative usage (odds ratio, 3.059; 95% confidence interval, 1.523–6.144; *p* = 2.0 × 10^−3^).

**Conclusion:**

Benzodiazepines may be associated with constipation in patients with schizophrenia. Therefore, clinicians should be cautious when prescribing benzodiazepines for the treatment of schizophrenia.

## Abstract 2

The results of univariate and multivariate logistic regression analyses in the laxative and non‐laxative groups are shown in graphical abstract. The results of multivariate logistic regression analyses revealed that benzodiazepine prescriptions were significantly higher in the laxative group (OR, 3.059; 95% CI, 1.523–6.144; *p* = 2.0 × 10^−3^).

## Graphical abstract

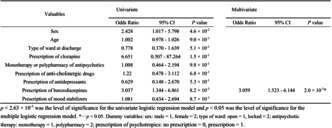

## 1 INTRODUCTION

Schizophrenia is a chronic and complex mental disorder characterized by positive and negative symptoms and cognitive impairment.^1^, ^2^ Antipsychotics are known to improve the symptoms of schizophrenia,^3^ and most clinical practice guidelines recommend antipsychotic monotherapy, especially using atypical antipsychotics, as a first‐line treatment because of their fewer adverse events such as extrapyramidal syndromes (EPS).^4^, ^5^, ^6^, ^7^, ^8^ Constipation is one of the adverse effects of psychotropics such as antipsychotics and anti‐cholinergic drugs.^5^ The prevalence of constipation in patients with schizophrenia is reportedly >30%.^9^, ^10^ Furthermore, only approximately 56% of the patients are aware of their constipation, and only 18.5% reported it to their doctors.^10^ Although constipation is highly prevalent in patients receiving treatment for schizophrenia, its symptoms may make it difficult for patients with schizophrenia to recognize it. Constipation can lead to more severe gastrointestinal diseases such as ileus.^11^ Furthermore, a recent study reported that constipation and laxative use are associated with mortality,^12^ and a recent meta‐analysis reported that chronic constipation is associated with an increased risk of all‐cause and cardiovascular disease‐related mortality.^13^ Thus, constipation may be an important complication and/or adverse effect that is also associated with these problems.

Although anti‐cholinergic drugs often induce constipation, several previous studies have examined the relationship of constipation only with antipsychotics and/or anti‐cholinergic drugs,^9^, ^10^, ^14^, ^15^, ^16^ suggesting that constipation in schizophrenia is associated with specific antipsychotics showing anti‐cholinergic effects, such as clozapine, and anti‐cholinergic drugs. In contrast, studies with aripiprazole and olanzapine in Asian patients with bipolar disorder showed no difference in constipation between the placebo and drug treatment groups.^17^

Although a recent meta‐analysis reported that long‐term benzodiazepine use significantly increased constipation in comparison with placebo in patients with anxiety disorders,^18^ no previous study has evaluated the differences in the provision of laxative treatment for schizophrenia in relation to benzodiazepines. Thus, studies on the relationship between constipation and psychotropic medications in schizophrenia are not consistent. To clarify the association between pharmacology and constipation in schizophrenia, the associations of psychotropic drugs other than antipsychotics and anti‐cholinergic drugs, such as benzodiazepines and mood stabilizers, also require investigation. Therefore, in this study, we investigated differences in the characteristics of pharmacotherapy for schizophrenia in relation to the prescription of laxatives at discharge.

## 2 METHODS

### 2.1 Patients

This cross‐sectional study was conducted in accordance with the Declaration of Helsinki, and information was collected from the medical records of patients at each institution with opt‐out consent. In this study, we collected data for the period from April 1st to September 30th, 2020, from eight institutions: Dokkyo Medical University, Ehime University, Fukuoka University, Jichi Medical University, Jikei University, Kitasato University, Kyorin University, and the University of Tokyo. We selected the patients who were diagnosed with schizophrenia according to the Diagnostic and Statistical Manual of Mental Disorders 5th edition (DSM‐5),^19^ and discharged from each university hospital for the period. We excluded patients who were not treated psychiatrically but only treated with physical illness and patients with serious physical complications or alcoholism. Using the medical records of the participating institutions, we recorded the discharge information of patients including data for age; sex; laxative prescription; all types and doses of prescribed psychotropics, including antipsychotics, antidepressants, anti‐cholinergic drugs, hypnotics and anxiolytics, mood stabilizers/antiepileptics, and other types of drugs; and the type of ward at discharge (open or locked).

Ethical approval was obtained from the ethics committees of Dokkyo Medical University, Ehime University, Fukuoka University, Jichi Medical University, Jikei University, Kitasato University, Kyorin University, and the University of Tokyo.

### 2.2 Procedure

The patients were divided into those who were prescribed laxatives (laxative group) and those who were not (non‐laxative group), and the findings for the two groups were compared. More specifically, we compared the mean numbers of different types of antipsychotics and the mean number of different types of psychotropics, including antipsychotics, prescribed to the patients in the two groups. We also compared the prescription rates of other psychotropic drugs, such as anti‐cholinergic drugs, antidepressants, benzodiazepines (anxiolytics and hypnotics), and mood stabilizers. In this study, lithium, valproate, carbamazepine, and lamotrigine were defined as mood stabilizers because they have been approved as mood stabilizers in Japan.

We converted the psychotropic dose into psychotropic dose equivalents, such as the chlorpromazine equivalent (CP), imipramine equivalent (IP), biperiden equivalent (BP), and diazepam equivalent (DP),^20^ and compared the prescribed doses of psychotropics.

Next, to clarify the influence of laxative use, we compared sex, age, type of ward at discharge (open or locked), and prescriptions of antipsychotics, clozapine, anti‐cholinergic drugs, antidepressants, benzodiazepines, and mood stabilizers as covariates using logistic regression with or without laxatives at discharge. As dummy variables, we defined sex: Male = 1, Female = 2; type of ward: Open = 1, Locked = 2; antipsychotic therapy: Monotherapy = 1, Polypharmacy = 2; prescription of psychotropics: no prescription = 0, prescription = 1.

### 2.3 Statistical analysis

Statistical analyses were performed using SPSS software (version 22.0; IBM Co., Armonk, NY, USA). The Shapiro–Wilk test was used to test for normality. The chi‐square test was used for categorical variables, and the Mann–Whitney *U* test was used for continuous and ordered variables to compare the two groups. We then calculated the adjusted odds ratios (ORs) with 95% confidence intervals (CIs) for the risk of constipation using a logistic regression model. We defined *p* < 2.63 × 10^−3^ (0.05/19) as significant, adjusted by Bonferroni's correction for post‐hoc analyses for multiple comparisons of categories, and defined *p* < 2.63 × 10^−3^ as significant for the univariate logistic regression model and *p* < 0.05 as significant for the multiple logistic regression model. Descriptive statistics were expressed as mean ± standard deviation (SD).

## 3 RESULTS

### 3.1 Characteristics of patients and prescriptions of psychotropics

The study population included 139 patients (62 in the laxative group and 77 in the non‐laxative group). The characteristics of the patients in the two groups are shown in Table 1. The two groups showed no significant differences in age, sex, or type of ward at discharge.

**TABLE 1:** Patient characteristics in relation to laxative prescription at discharge.

	Patients prescribed laxatives (*n* = 62)	Patients not prescribed laxatives (*n* = 77)	*p* value
Female (%)	80.6 (50)	63.6 (49)	2.8 × 10^−2^
Age (years)	43.0 ± 16.1	42.9 ± 14.6	9.7 × 10^−1^
Type of ward at discharge (Open: Locked)	32:30	34:43	3.8 × 10^−1^
Mean number of all types of laxatives prescribed at discharge (*N*/day)	1.6 ± 0.9	Not Applicable	Not Applicable

*Note*: Values are expressed as mean ± SD. Comparison between patients with and without laxative prescriptions was performed using the chi‐squared test or Mann–Whitney *U* test with Bonferroni correction. *p* < 2.63 × 10^‐3^ as significance.

The prescription rates of psychotropics at discharge in the two groups are shown in Table 2. The benzodiazepine prescription rate in the laxative group was significantly higher than that in the non‐laxative group (66.1% vs. 39.0%; *p* = 1.4 × 10^−3^), and the mean number of benzodiazepines in the laxative group was significantly higher than that in the non‐laxative group (1.2 ± 1.1/day vs. 0.7 ± 0.9/day; *p* = 2.6 × 10^−3^). The other variables showed no significant differences between the two groups.

**TABLE 2:** Prescription of psychotropics at discharge.

	Patients prescribed laxatives (*n* = 62)	Patients not prescribed laxatives (*n* = 77)	*p* value
Prescription (%)			
Antipsychotics	100 (62)	98.7 (76)	5.5 × 10^−1^
Antipsychotic monotherapy	53.2 (33)	63.2 (48)	2.8 × 10^−1^
Clozapine	3.2 (2)	1.3 (1)	4.4 × 10^−1^
Anti‐cholinergic drugs	29.0 (18)	16.9 (13)	8.7 × 10^−2^
Antidepressants	6.5 (4)	7.8 (6)	7.6 × 10^−1^
Benzodiazepines	66.1 (41)	39.0 (30)	1.4 × 10^−3^*
Mood stabilizers	30.6 (19)	18.2 (14)	8.6 × 10^−2^
Number of psychotropics (*N*/day)			
Antipsychotics	1.6 ± 0.8	1.4 ± 0.6	1.3 × 10^−1^
Anti‐cholinergic drugs	0.4 ± 0.6	0.2 ± 0.4	1.0 × 10^−1^
Antidepressants	0.1 ± 0.2	0.1 ± 0.3	7.6 × 10^−1^
Benzodiazepines	1.2 ± 1.1	0.7 ± 0.9	2.6 × 10^−3^*
Mood stabilizers	0.1 ± 0.2	0.1 ± 0.3	5.6 × 10^−1^
Mean dose of psychotropics (mg/day)			
Antipsychotics^a^	777.9 ± 476.7	554.4 ± 376.4	3.4 × 10^−3^
Anti‐cholinergic drugs^b^	3.1 ± 2.2	2.4 ± 0.8	6.5 × 10^−1^
Antidepressants^c^	115.6 ± 129.7	41.7 ± 29.2	2.8 × 10^−1^
Benzodiazepines^d^	16.4 ± 15.4	12.9 ± 12.7	1.7 × 10^−1^

*Note*: Values are expressed as mean ± SD. Comparison between patients with and without laxative prescriptions was performed using the chi‐squared test or Mann–Whitney *U* test with Bonferroni correction. *p < 2.63 × 10^‐3^ indicated significance.

^a^ Presented as chlorpromazine equivalent.

^b^ Presented as biperiden equivalent.

^c^ Presented as imipramine equivalent.

^d^ Presented as diazepam equivalent.

### 3.2 The results of multivariate logistic regression analyses associated with laxative prescription

The results of univariate and multivariate logistic regression analyses in the laxative and non‐laxative groups are shown in Table 3. The results of multivariate logistic regression analyses revealed that benzodiazepine prescriptions were significantly higher in the laxative group (OR, 3.059; 95% CI, 1.523–6.144; *p* = 2.0 × 10^−3^).

**TABLE 3:** Multiple logistic regression models of laxative prescription at discharge.

Valuables	Univariate			Multivariate		
	Odds ratio	95% CI	*p* value	Odds ratio	95% CI	*p* value
Sex	2.428	1.017–5.798	4.6 × 10^−2^			
Age	1.002	0.978–1.026	9.0 × 10^−1^			
Type of ward at discharge	0.778	0.370–1.639	5.1 × 10^−1^			
Prescription of clozapine	6.651	0.507–87.264	1.5 × 10^−1^			
Monotherapy or polypharmacy of antipsychotics	1.008	0.464–2.194	9.8 × 10^−1^			
Prescription of anti‐cholinergic drugs	1.22	0.478–3.112	6.8 × 10^−1^			
Prescription of antidepressants	0.629	0.148–2.670	5.3 × 10^−1^			
Prescription of benzodiazepines	3.037	1.344–6.861	8.2 × 10^−3^	3.059	1.523–6.144	2.0 × 10^−3^*
Prescription of mood stabilizers	1.081	0.434–2.694	8.7 × 10^−1^			

*Note*: *p* < 2.63 × 10^−3^ was the level of significance for the univariate logistic regression model and *p* < 0.05 was the level of significance for the multiple logistic regression model. *p < 0.05. Dummy variables: sex: male = 1, female = 2; type of ward: open = 1, locked = 2; antipsychotic therapy: monotherapy = 1, polypharmacy = 2; prescription of psychotropics: no prescription = 0, prescription = 1.

## 4 DISCUSSION

In this study, we observed a significant association between laxative use and benzodiazepine prescriptions in patients with schizophrenia. However, we did not find a significant association between laxative use and prescriptions of anti‐cholinergic drugs. Although some studies have examined the association between schizophrenia and laxatives and/or constipation,^9^, ^10^, ^14^, ^15^, ^16^ to the best of our knowledge, this is the first study to report a significant association between laxative use and benzodiazepine prescription in patients with schizophrenia. This result suggests that benzodiazepine prescriptions would be associated with constipation and the use of laxatives and that benzodiazepine prescriptions for the treatment of schizophrenia should be performed after careful consideration.

In the present study, the prescription rate of benzodiazepines in the laxative group was 66.1%, making benzodiazepines the most frequently prescribed concomitant psychotropics in this study. In Asia, the prescription rate of benzodiazepines was the second‐highest among concomitant psychotropic drugs, and was second only to anti‐cholinergic drugs, in 2016.^21^ In Europe, the prescription rate of benzodiazepines was approximately 30%, even in older patients with schizophrenia spectrum disorders.^22^ In addition to constipation, high exposure to benzodiazepines, but not antipsychotics and antidepressants, was significantly associated with mortality in patients with schizophrenia.^23^ Thus, a higher prescription rate of benzodiazepines is a worldwide psychopharmacological problem in the treatment of schizophrenia. We recently reported that psychiatrist participation in educational programs on clinical guidelines was significantly associated with higher non‐prescription of anxiolytics or hypnotics for schizophrenia than the corresponding rates among psychiatrists who did not participate in these programs.^24^ Thus, a guideline‐based education program may be useful in decreasing the prescription rate of benzodiazepines for schizophrenia.

Previous studies on psychiatric disorders other than schizophrenia^18^, ^25^, ^26^, ^27^ did not discuss the mechanisms underlying the association between constipation and benzodiazepine use. For anxiety or insomnia, the effects of benzodiazepines are primarily associated with the gamma‐aminobutyric acid (GABA) receptors in the brain, especially GABA_A_ receptors.^28^ GABA_A_ receptors also exist in the colonic neurons and play an important role in the regulation of gastrointestinal activity.^29^, ^30^, ^31^ In in vivo studies, GABA_A_ receptor subtypes were shown to regulate stress‐induced colon inflammation in the early life stress model.^32^ Thus, complex interactions between GABA and the gastrointestinal tract may play an important role in the pathogenesis of constipation.

However, in this study, we did not observe a significant association between constipation and the use of anti‐cholinergic drugs, unlike the findings reported in previous studies. Lin et al.^14^ reported a significant association between constipation and clozapine, the dose of antipsychotics, and anti‐cholinergic drugs. In the present study, the overall prescription rate of anti‐cholinergic drugs was 22.3%, and the rate in the laxative group was 29.0%. This proportion was lower than that reported in previous studies.^10^, ^14^ In Europe, the prescription rate for anti‐cholinergic drugs is low.^33^ Virtanen et al.^9^ reported an association between constipation and antipsychotics, but not anti‐cholinergic drugs. Furthermore, Lin reported that older patients with schizophrenia did not show a relationship between constipation and anti‐cholinergic drugs, but showed significant correlations between constipation and some antipsychotics and mood stabilizers in the same institutions.^16^ Thus, these results suggest that constipation in schizophrenia may not be directly caused by the anti‐cholinergic effect, but may be mediated by complex mechanisms, including the effects of other drugs.

In this study, the mean dose of antipsychotics in the laxative group tended to be higher than that in the non‐laxative group; however, the difference was not significant. Specific antipsychotics, such as clozapine, have been suggested to be associated with constipation^9^; however, the association with dose in previous studies has been inconsistent,^14^, ^15^, ^16^ and the mechanism is still unclear. Nevertheless, the number of patients prescribed clozapine in this study was small; thus, future studies with larger sample sizes are required.

This study had several limitations. First, because all participants in this study were discharged from university hospitals, there may have been a selection bias. Second, the sample size was relatively small. However, we recruited participants from rural and urban areas in Japan, so bias attributable to factors such as regional characteristics may have been lower. Third, since we only obtained data at the time of discharge, the duration of benzodiazepine use in the patients could not be investigated. Fourth, this was a retrospective, observational study. Prospective studies and randomized controlled trials are needed to further investigate the association between constipation and benzodiazepines in patients with schizophrenia. Fifth, clinicians may have prescribed laxatives unnecessarily for a long time and, in that case, laxative use cannot be an indicator of constipation. In this study, we could not distinguish whether laxatives were used as the appropriate use for constipation.

In conclusion, benzodiazepines may be associated with constipation in patients with schizophrenia. When clinicians observe constipation in patients with schizophrenia, they should consider not only the dose of antipsychotics and anti‐cholinergic drugs, but also prescriptions of benzodiazepines and consider reducing or discontinuing benzodiazepines.

## AUTHOR CONTRIBUTIONS

**Shinichiro Ochi:** Writing—original draft, Writing—review & editing, Investigation, Software, Visualization, Resources, Formal analysis, Data curation. **Takashi Tsuboi:** Writing—review & editing, Visualization, Investigation, Software, Resources, Project administration, Methodology, Formal analysis, Validation. **Naomi Hasegawa:** Writing—review & editing, Resources. **Hikaru Hori:** Writing – review & editing, Visualization, Investigation, Resources, Project administration, Methodology, Formal analysis, Conceptualization. **Kayo Ichihashi:** Writing—review & editing, Investigation, Resources. **Yayoi Imamura:** Writing—review & editing, Investigation, Resources. **Tsuyoshi Okada:** Writing—review & editing, Investigation, Resources. **Fumitoshi Kodaka:** Writing—review & editing, Investigation, Resources. **Yoshitaka Saito:** Writing—review & editing, Investigation, Resources. **Jun‐ichi Iga:** Writing—review & editing, Visualization, Investigation. **Toshiaki Onitsuka:** Writing—review & editing, Investigation. **Kiyokazu Atake:** Writing—review & editing. **Shu‐ichi Ueno:** Writing—review & editing, Supervision. **Ryota Hashimoto:** Writing—review & editing, Visualization, Project administration, Methodology, Supervision, Funding acquisition, Conceptualization. **Norio Yasui‐Furukori:** Writing—review & editing, Visualization, Investigation, Resources, Project administration, Methodology, Supervision, Conceptualization.

## FUNDING INFORMATION

This study was supported by the Japan Agency for Medical Research and Development (AMED) (Grant Numbers JP16dk0307060, JP19dk0307083, and JP22dk0307112), JSPS KAKENHI (Grant Numbers JP21K17261 and JP23K16273), Health and Labor Sciences Research Grants (Grant Numbers H29‐Seishin‐Ippan‐001 and 19GC1012), the Japanese Society of Neuropsychopharmacology, the Japanese Society of Mood Disorders, the Japanese Society of Clinical Neuropsychopharmacology and the Japanese Society of Psychiatry and Neurology. The funders had no role in the study design, data collection and analysis, decision to publish, or preparation of the manuscript.

## CONFLICT OF INTEREST STATEMENT

The authors declare no conflict of interest. Norio Yasui‐Furukori, Hikaru Hori, and Jun‐Ichi Iga are Editorial Board member of Neuropsychopharmacology Reports. Hikaru Hori is also a co‐author of this article.

## ETHICAL APPROVAL

Approval of the Research Protocol by an Institutional Reviewer Board: This study was approved by the ethics committees of Dokkyo Medical University (R‐61‐2J), Ehime University (2022A019), Fukuoka University (U22‐10‐010), Jichi Medical University (22‐090), Jikei University (36‐020(12119)), Kitasato University (C22‐057), Kyorin University (R04‐002), and the University of Tokyo (2022137NI).

Informed Consent: We obtained information from the medical records with opt‐out consent.

Registry and the Registration No. of the Study/Trial: N/A.

Animal Studies: N/A.

## Data Availability

The data are not publicly available due to privacy and ethical restrictions (i.e., we did notobtain informed consent on the public availability of raw data).
